# Draft genome sequence data of a clinical *Enterococcus faecalis* isolate SHH039 from a patient with cholecystitis from a tertiary care hospital in Sabah, Malaysia

**DOI:** 10.1016/j.dib.2022.108019

**Published:** 2022-03-06

**Authors:** Nur Nashyiroh Izayati Mastor, Vijay Kumar Subbiah, Wan Nazirah Wan Abu Bakar, Khurshida Begum, M. Jahangir Alam, Mohammad Zahirul Hoque

**Affiliations:** a Department of Pathobiology & Medical Diagnostics, Faculty of Medicine and Health Sciences, University Malaysia Sabah, Sabah, Malaysia; b Biotechnology Research Institute, University Malaysia Sabah, Sabah, Malaysia; c Department of Pathology, Queen Elizabeth Hospital, Ministry of Health Malaysia, Kota Kinabalu, Sabah, Malaysia; d Department of Pharmacy Practice and Translational Research, University of Houston College of Pharmacy, Houston, Texas, USA

**Keywords:** *Enterococcus faecalis*, Cholecystitis, Antibiotic sensitivity and resistance, Draft genome sequencing Sabah, Malaysia

## Abstract

An *Enterococcus faecalis* strain SHH039 was isolated from a 68 year old man who was hospitalised with cholecystitis. The genomic sequence of this isolate which had a size of 2,990,081 bp and 2,663 proteins with functional assignments is presented here. Analysis of the genome revealed *Enterococcus faecalis* with multiple antibiotic resistance genes which may be associated with acute cholecystitis. It may be not clear if the infection symptoms are the consequence of enterococci manifestation. However, this opportunistic organism may play a minor role in the disease.

## Specifications Table

**Table:** 

Subject	Microbiology
Specific subject area	Genomic.
Type of data	Data related to genomic sequence and antimicrobial resistance.
How data were acquired	Whole genome was sequenced with an Illumina HiSeq 4000 sequencing platform.
Data format	Raw and analyzed.
Parameters for data collection	*Enterococcus faecalis* strain SHH039 was isolated from the gallbladder bile of a 58-year-old male patient with acute cholecystitis.
Description of data collection	*Enterococcus faecalis* strain SHH039 was sequenced with Illumina HiSeq 4000 platform and core-SNP based phylogenetic tree was then constructed with other 20 clinical enterococcal isolates extracted from the GenBank public database.
Data source location	Male Medical Ward of the Queen Elizabeth Hospital, Kota Kinabalu, Sabah, Malaysia Borneo
Data accessibility	Data is available in NCBI repository. This whole-genome project has been deposited in DDBJ/ENA/GenBank under accession no. JAEFCX000000000. (https://www.ncbi.nlm.nih.gov/nuccore/JAEFCX000000000.1). The version described in this paper is the first version (JAEFCX000000000). The raw sequencing reads have been deposited in the Sequence Read Archive (SRA) under accession No. SRR13153714. (https://www.ncbi.nlm.nih.gov/sra/SRR13153714).

## Value of the Data

• The draft genome sequence data analysis revealed the presence of *Enterococcus faecalis* strain SHH039 which was isolated from a male patient with acute cholecystitis in a local tertiary hospital in Kota Kinabalu, Sabah.
• The data is important to understand the presence of *Enterococcus faecalis* in polymicrobial bacteraemia and may be the aetiology of acute cholecystitis in hospitalized patients as well as if it belongs to the same group as the other human clinical enterococcal infections.
• The antibiotic sensitivity and resistance data of *Enterococcus faecalis* will be useful and potentially help to establish new therapies for variety of enterococcal infections and might reveal insights into these roles.

## 1 Data Description

**Fig. 1:**
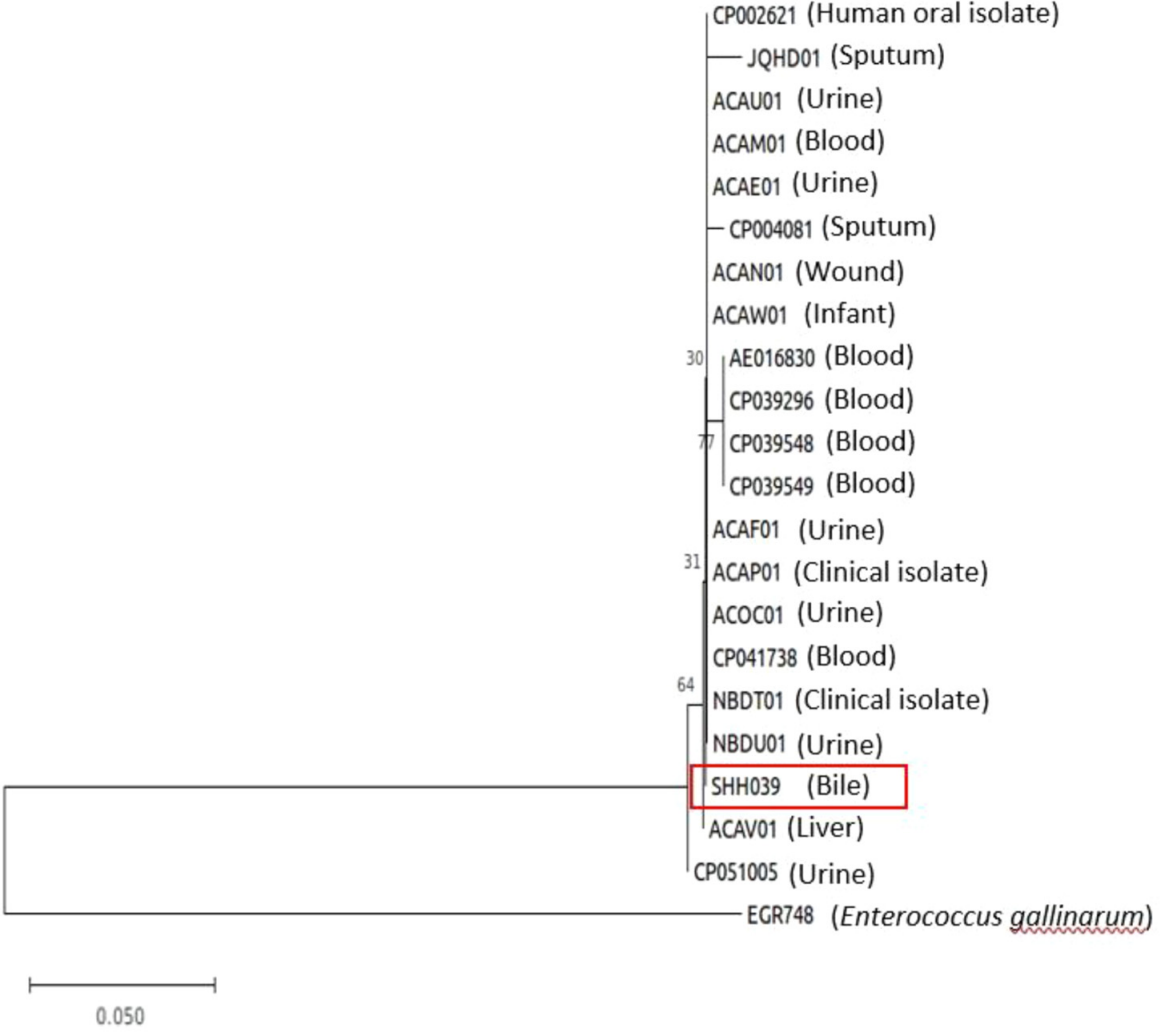
Phylogenetic tree illustrating the position of *Enterococcus faecalis* strain SHH039 based on the core-SNP sequences in relation to other 20 clinical *Enterococcus faecalis* extracted from the GenBank public database and its accession number. The tree was built using MEGA 8.0 with 1000 bootstrap iterations.

*Enterococcus* species are commensal inhabitants of the gastrointestinal tract in healthy individuals. Two species under this genera, *Enterococcus faecium* and *Enterococcus faecalis*, has a high-level resistance to multiple antibiotics and an active transmission in hospitals and communities [1]. The most common symptoms associated with *Enterococcus* are endocarditis, urinary tract infections (UTIs), wound infections, sepsis and bacteraemia. However, in the last few years, the emergence of multidrug-resistant enterococci strains has been reported from intra-abdomen or biliary sources indwelling central lines, or soft tissue infections [2,3]. It is found to be more prevalent as a component of polymicrobial bacteraemia than other pathogens such as *Escherichia coli* and other gram-negative bacilli [4], [5], [6], [7]. The draft genomic sequence of *Enterococcus faecalis* SHH039, which was isolated from the gallbladder bile of a hospitalised male patient with acute cholecystitis in July 2020, is presented here. A phylogenetic tree was generated usingthe core-SNP genomes of these isolates and the genomes of another 20 genomes clinical enterococcal isolates (Fig. 1) obtained from the GenBank public database. The susceptibility tests with the Vitek 2 (bioMérieux, Inc., Durham, NC) system were performed using software version 5.01 and AST-GP2 (enterococci) cards according to the manufacturer's protocol showed that the isolate was ampicillin, gentamycin, nitrofurantoin, vancomycin and linezolid sensitive but resistant to tetracycline (Supplementary Table 1). The *Enterococcus faecalis* SHH039 draft genome is 2,990,081 bp assembled in 45 contigs and has a GC content of 37.30%. The genome coverage was 300x and the N50 score was 270,652 bp. The PGAP annotation identified 2,663 protein-coding genes, 52 tRNAs and 7 complete rRNAs (one 5S rRNAs, two 16S rRNA, and four 23S rRNA) (Table 1).

**Table 1:** Statistics of assembled sequence of *Enterococcus faecalis* SHH039.

Assembly Statistic	Final Assembly
Total sequence read	7,085,168
Sequencing Depth	300x
No of contigs	45
Total sequence length (bp)	2,990,081
Minimum sequence length (bp)	78
Maximum sequence length (bp)	750,409
N50(bp)	270,652
GC content (%)	37.30%
CDSs	2,663
tRNAs	52
5s,16s,23s rRNA	1,2,4

## 2 Experimental Design, Materials and Methods

### 2.1 Bacteria strain isolation, DNA extraction, library preparation and sequencing

The *Enterococcus faecalis* strain SHH039 was isolated from a 68 year old male patient's gallbladder bile who was hospitalized with acute cholecystitis in July 2020. The isolate was streaked onto blood agar and incubated at 37 °C for 24 h. A single pure culture from the overnight blood agar plate was inoculated on Luria Bertani (LB) broth High Salt (Hazelwood, Mo) and incubated again overnight. The fully automated VITEK2 (BioMérieux, Inc, Hazelwood, Mo) technology was initially utilised to identify the bacterial species and subsequently confirmation was done using 16S rRNA Sequencing. We used the the BigDye Terminator (v3.1) on a ABI3130 DNA Sequencer (Applied Biosystem, USA). The Qiagen DNeasy blood and tissue kit (Qiagen, Valencia, CA) was used to extract the genomic DNA according to the manufacturer's protocol. The Nanodrop 2000c spectrophotometer (ThermoFisher Scientific, USA) and Qubit® 2.0 fluorometer (Invitrogen, ThermoFisher Scientific, USA) were used to quantify the DNA concentrations. Finally, the NEBnext Ultra kit (New England Biolabs, NEB #E7645) were used to prepare the Whole genome-sequencing libraries and then sequenced with the Illumina HiSeq 4000 platform.

### 2.2 Genome sequencing, assembly, annotation and screening of antimicrobial resistance gene

Using Trimmomatic version 0.39 [8], all raw reads were pre-processed, adapters were trimmed and reads with fewer than 50 bp were eliminated, based on phred with a quality below Q30. Next the genome was assembled with SPAdes version 3.11.1 [9] and annotated with PGAP (Prokaryotic Genome Annotation Pipeline) [10]. Using the Comprehensive Antibiotic Resistance Database (CARD) version 3.0.3 tools [11], the antimicrobial resistance gene in *Enterococcus faecalis* strain SHH039 identified were including dfrE (encoding for dihydrofolate reductase), efrA (encoding for ATP-binding cassette-ABC efflux pump) and tet(M) (associated with tetracycline resistance). The data sequence was deposited in the Sequence Read Archive (SRA) under the bioproject accession number PRJNA680852 (Biosample accession number SAMN16925233).

### 2.3 Phylogenetic tree analysis

A phylogenetic tree was reconstructed based on the comparative sequence analysis of the core-SNP genomes of these isolates with 20 genomes clinical enterococcal isolates extracted from the GenBank public database (Fig. 1). The core-SNP sequences were aligned using kSNP3 tools based on k-mer analysis [12] and phylogenetic inferences were obtained using the maximum-likelihood method in MEGA (Molecular Evolutionary Genetic Analysis) software 8.0 package [13]. Bootstrap analysis of 1000 replicates was used to evaluate the significance of the branching patterns.

## Nucleotide Sequence Accession Number

This whole-genome project has been deposited in DDBJ/ENA/GenBank under accession no. JAEFCX000000000. The version described in this paper is the first version (JAEFCX000000000). The raw sequencing reads have been deposited in the SRA under accession no. SRR13153714.

## Ethics Statement

This research was approved by the Medical Research Ethics Committee (MREC), Ministry of Health, Malaysia (No. NMRR-19-1770–48622) and Universiti Malaysia Sabah Medical Ethics Committee (No. JKEtika 1/19(26).

## CRediT Author Statement

**Nur Nashyiroh Izayati Mastor:** Methodology, Data curation, Formal analysis, Investigation, Software, Writing – Original draft preparation; **Vijay Kumar Subbiah:** Conceptualization, Data curation, Investigation, Methodology, Writing - Reviewing and Editing, Supervision, Funding acquisition; **Wan Nazirah Wan Abu Bakar:** Data curation, Investigation; **Khurshida Begum:** Methodology, Investigation; **M. Jahangir Alam:** Conceptualization, Writing - Reviewing and Editing, Validation, Methodology; **Mohammad Zahirul Hoque:** Conceptualization, Data curation, Methodology, Formal Analysis, Funding acquisition, Writing - Reviewing and Editing, Supervision.

## Declaration of Competing Interest

The authors declare that they have no known competing financial interests or personal relationships that could have appeared to influence the work reported in this paper.

## Appendix Supplementary materials

Image, application 1
