# Supplementary material for: Draft genome sequence data of a clinical Enterococcus faecalis isolate SHH039 from a patient with cholecystitis from a tertiary care hospital in Sabah, Malaysia
Source: Data Brief. 2022 Mar 6;41:108019. doi: 10.1016/j.dib.2022.108019 (PMC8919231; doi:10.1016/j.dib.2022.108019)
Supplement: Supplementary file 1 [file mmc1.docx]

Supplementary Table 1: Antibiotic susceptibility data for *Enterococcus faecalis* SHH039

| Antibiotics | Sensitive | Resistant | MIC value |
| --- | --- | --- | --- |
| Ampicillin |  |  | <=2 |
| Gentamicin |  |  | SYN-R |
| Nitrofurantoin |  |  | <=16 |
| Linezolid |  |  | 2 |
| Vancomycin |  |  | 1 |
| Tetracycline |  |  | >=16 |

*MIC = Minimum Inhibitory Concentrations

MIC Interpretation Guideline: Global CLSI-based (2019)

AES Parameter Set Name: Global CLSI-based+Natural Resistance (2019)
